# Risk factors for renal failure and short-term prognosis in patients with spontaneous intracerebral haemorrhage complicated by acute kidney injury

**DOI:** 10.1186/s12882-020-01949-9

**Published:** 2020-07-29

**Authors:** Zhenhuan Zou, Siying Chen, Yinshuang Li, Jiawei Cai, Yulu Fang, Jingzhi Xie, Wenhua Fang, Dezhi Kang, Yanfang Xu

**Affiliations:** 1 Department of Nephrology, First Affiliated Hospital, Fujian Medical University, Chazhong Road 20, Fuzhou, 350005 China; 2 Department of Neurosurgery, First Affiliated Hospital, Fujian Medical University, Fuzhou, 350005 China

**Keywords:** Spontaneous intracerebral haemorrhage, Acute kidney injury, Risk factors, Short-term prognosis

## Abstract

**Background:**

Although acute kidney injury (AKI) is a known risk factor for adverse clinical outcomes in patients with spontaneous intracerebral haemorrhage (SICH), little is known about the predisposing factors that contribute to renal failure and short-term prognosis in the setting of SICH already complicated by AKI. In this study, we aimed to identify the renal failure factors in SICH patents with AKI.

**Methods:**

Five hundred forty-three patients with SICH complicated by differential severities of AKI who were admitted to the First Affiliated Hospital of Fujian Medical University from January 2016 to December 2018 were retrospectively studied. Logistic regression and receiver operator characteristic (ROC) curve analysis were performed to determine the best predictive and discriminative variables. Multivariate Cox regression analysis was performed to identify prognostic factors for renal recovery.

**Results:**

In the multivariable adjusted model, we found that hypernatremia, metabolic acidosis, elevated serum creatine kinase, hyperuricaemia, proteinuria, and the use of colloids and diuretics were all independent risk factors for the occurrence of stage 3 AKI in SICH patients. The area under the curve analysis indicated that hypernatremia and hyperuricaemia were predictive factors for stage 3 AKI, and the combination of these two parameters increased their predictability for stage 3 AKI. Kaplan-Meier survival curves revealed that the renal recovery rate in SICH patients with stages 1 and 2 AKI was significantly higher than that in SICH patients with stage 3 AKI. Multivariate Cox regression analysis suggested that hypernatremia and the occurrence of stage 3 AKI are predictors for poor short-term renal recovery.

**Conclusions:**

These findings illustrate that hypernatremia and hyperuricaemia represent potential risk factors for the occurrence of stage 3 AKI in SICH patients. Those patients with hypernatremia and stage 3 AKI were associated with a poor short-term prognosis in renal recovery.

## Background

Acute kidney injury (AKI), characterized by the sudden deterioration of kidney function or kidney damage occurring over hours to a few days, is a common complication following both acute ischaemic stroke (AIS) and intracerebral haemorrhage (SICH) [1]. While AKI has been reportedly associated with increased mortality following AIS [2–5] and SICH [1, 6], SICH patients are at risk of AKI complicated by hypertension, diabetes mellitus and atherosclerosis. The inappropriate therapies also increase the risk of AKI in SICH patients. In spite of these facts, the association between AKI and SICH remains poorly explored as very few studies have addressed this [2, 5–8]. Most of these previous studies have utilized 3 strata of severity (risk, injury and failure) based on the magnitude of increase in serum creatinine level and/or the duration of oliguria,as well as 2 outcome stages (loss of kidney function and end-stage kidney disease) or different standards of classifications for defining AKI [9]. A most recent study by Ansaritoroghi et al. according to the new Kidney Disease: Improving Global Outcomes (KDIGO) criteria [10, 11], which is now generally accepted as a highly sensitive definition for AKI, as shown in determination of the incidence and risk factors for AKI in SICH patients and examination of the role of AKI on SICH mortality in a South Indian population. The authors found that reduced estimated glomerular filtration rate at admission and infections represent potential risk factors for the occurrence of AKI in SICH patients. Those patients with AKI were associated with poor neurological outcome and higher mortality,which increased with the severity of AKI [1]. However, to the best of our knowledge, there have been no studies published in the literature focusing on identification of risk and prognostic factors for the occurrence of a more severe stage of AKI defined by KDIGO criteria in patients after an episode of SICH. Hence, we conducted this retrospective study aimed at identifying both risk factors for the occurrence of stage 3 AKI in patients with SICH and prognostic factors for short-term renal recovery from AKI using the new KDIGO guidelines.

## Methods

### Study population

This was a retrospective study of 543 SICH patients with AKI complication who were admitted to the First Affiliated Hospital of Fujian Medical University from January 1, 2016 to December 31, 2018. SICH was diagnosed based on the American Heart Association/American Stroke consensus criteria [12]. The definition and staging of AKI were based on the serum creatinine (Scr) criteria according to Kidney Disease: Improving Global Outcomes (KDIGO) guidelines in 2012 [10]. The following exclusion criteria were applied: patients with pre-existing chronic kidney disease (CKD), age below 16, and duration of hospitalization less than 2 days.

### Definition and staging of AKI

For this study, the diagnosis of AKI was described as follows [10]: an increase in SCr by ≥0.3 mg/dL (≥26.5 μmol/l) within 48 h or an increase in SCr to ≥1.5 times baseline, which is known or presumed to have occurred within the 7 days prior. AKI is staged for severity according to the following criteria [10]: stage 1, an increase in SCr of ≥0.3 mg/dL (≥26.5 μmol/l) or an increase in SCr by 1.5–1.9 times baseline; stage 2, an increase in SCr by 2.0–2.9 times baseline; and stage 3, an increase in SCr to 4.0 mg/dL (353.6 μmol/l) or an increase in SCr by 3.0 times baseline. Refer to Ansaritoroghi’s study [1], urine output was not used for definition and staging of AKI in the study, because it was not routinely recorded in all patients on an hourly basis. Based on the AKI staging status, 543 cases were divided into two groups with stage 1 and 2 as the Injury group (*n* = 410) and stage 3 as the Failure group (*n* = 133).

### Clinical data

We retrospectively reviewed the computerized medical records at our hospital to obtain all patient data pertaining to medical histories, demographics characteristics, laboratory parameters, and drug use history. Laboratory data included serum levels of sodium, chloride, albumin, bicarbonate, creatine kinase and uric acid, as well as haemoglobin levels and white blood cell counts. Hypernatremia and hyperchloremia were diagnosed when plasma sodium and chloride levels were above 145 mmol/L and 108 mmol/L, respectively. Patients were considered to have hypoproteinemia and metabolic acidosis when plasma levels of albumin and bicarbonate were below 40 g/L and 22 mmol/L, respectively. Elevated serum creatine kinase was defined as a plasma level of creatine kinase above 310 U/L for males or 220 U/L for females. Hyperuricaemia was defined as a plasma level of uric acid above 7 mg/dl in males or 6 mg/dl in females. Anaemia was diagnosed when haemoglobin was below 130 g/L in males or 120 g/L in females. White blood cell counts above 9.5 × 10^9^/L were defined as elevated.

### Evaluation of short-term prognosis

In addition to assessing the occurrence of AKI after SICH onset and risk factors for the occurrence of stage 3, we evaluated short-term prognosis by monitoring renal function recovery over a 14-day period after an episode of SICH with AKI. As a terminal event, renal recovery was defined as a reduction in serum creatinine (SCr) of < 1.5 × baseline [13].

### Statistical analysis

We used SPSS version 25.0 for Windows (Chicago, IL, USA) to perform statistical analysis. Data are expressed as the median with the 25th and 75th quartiles for skewed data or as the mean ± SD for normally distributed data. Differences between groups were evaluated by a non-parametric test or Student’s *t*-test. Percentages were compared using the chi-squared test. A multivariate logistic regression forward stepwise model was used to identify independent risk factors for SICH complicated to stage 3 AKI. Forest plots were created using GraphPad Prism. Receiver operating characteristic (ROC) curve analysis was performed to determine the optimal cut-off values for predicting the occurrence of stage 3 AKI. Kaplan-Meier curve and log-rank tests were used to compare the rates of renal recovery between the Injury and Failure groups. Multivariate Cox regression analysis was performed to identify prognostic factors for renal recovery. In all analyses, *p*-values < 0.05 were considered statistically significant.

## Results

### Characteristics of study patients in the injury and failure groups

Demographic data, medical history, medications used and laboratory data are displayed in Table 1. Of 543 SICH patients complicated by AKI, 410 (75.5%) developed stage 1 and 2 AKI, while 133 (24.5%) were found to have stage 3 AKI. Mean age was 58.0 ± 15.2 years, and the population was predominantly male (66.9%, 363/453). The two groups (injury and failure) had similar ratios of age, gender and medical history with respect to diabetes and hypertension. The medications being used by the two groups were found to be statistically significant with the exception of aminoglycosides and NSAIDs. Compared to the injury group, a significant number of failure group patients exhibited a higher ratio of ICU admission and received mechanical ventilation (both *P* < 0.05). Levels of serum potassium, sodium and uric acid were all higher, and levels of serum bicarbonate and haemoglobin were lower in failure group patients (all *P* < 0.05). No significant differences were observed in serum levels of chloride, albumin, creatine kinase, or white blood cell count (all *P* > 0.05).

**Table 1:** Characteristics of the study patients in the Injury group and in the Failure group

Characteristics	Injury group	Failure group	*P* value
**Number (%)**	410 (75.5)	133 (24.5)	
**Demographics**			
Age (years)	58.4 ± 14.8	56.6 ± 16.2	0.219
Male, N (%)	270 (65.9)	93 (69.9)	0.386
**Medical history, N (%)**			
Diabetes	65 (15.9)	30 (22.6)	0.077
Hypertension	234 (57.1)	80 (60.2)	0.532
ICU admission	261 (63.6)	98 (73.6)	**0.034**
Mechanical ventilation	215 (52.4)	98 (73.6)	**< 0.001**
**Medication used**, **N (%)**			
Mannitol	295 (72)	97 (62.6)	**0.031**
NSAID	147 (35.9)	46 (34.6)	0.791
Colloids	259 (63.2)	105 (78.9)	**0.001**
Vasoactive drugs	44 (32.4)	89 (21.9)	**0.014**
Diuretics	281 (70.8)	129 (88.4)	**< 0.001**
Aminoglycosides	49 (12)	19 (14.3)	0.48
**Laboratory data**			
K^+^ (mmol/L)	3.97 ± 1.21	4.33 ± 1.08	**0.002**
Na^+^ (mmol/L)	145.55 ± 10.41	151.80 ± 15.62	**< 0.001**
Cl^−^ (mmol/L)	109.83 ± 48.60	113.36 ± 14.71	0.195
HCO_3_^−^ (mmol/L)	25.30 ± 5.60	23.51 ± 6.39	**0.002**
Alb (g/L)	37.54 ± 7.00	37.22 ± 8.02	0.689
CK (U/L)	441.63 ± 963.52	674.31 ± 1338.27	0.065
UA (mmol/L)	323.96 ± 147.08	439.79 ± 169.98	**< 0.001**
WBC (×10^9^/L)	12.29 ± 5.62	12.59 ± 5.22	0.579
HGB (g/L)	118.46 ± 26.14	111.19 ± 32.08	**0.019**

*ICU admission* intensive care unit admission, *K*^*+*^ serum potassium concentration, *Na*^*+*^ serum sodium concentration, *Cl*^−^ serum chloride concentration, *HCO*_*3*_^*−*^ serum bicarbonate concentration, *Alb* serum albumin concentration, *CK* serum creatine kinase, *UA* serum uric acid concentration, *WBC* white blood cell count, *HGB* hemoglobin

### Evaluation of risk factors for the occurrence of stage 3 AKI in patients with SICH

**Fig. 1:**
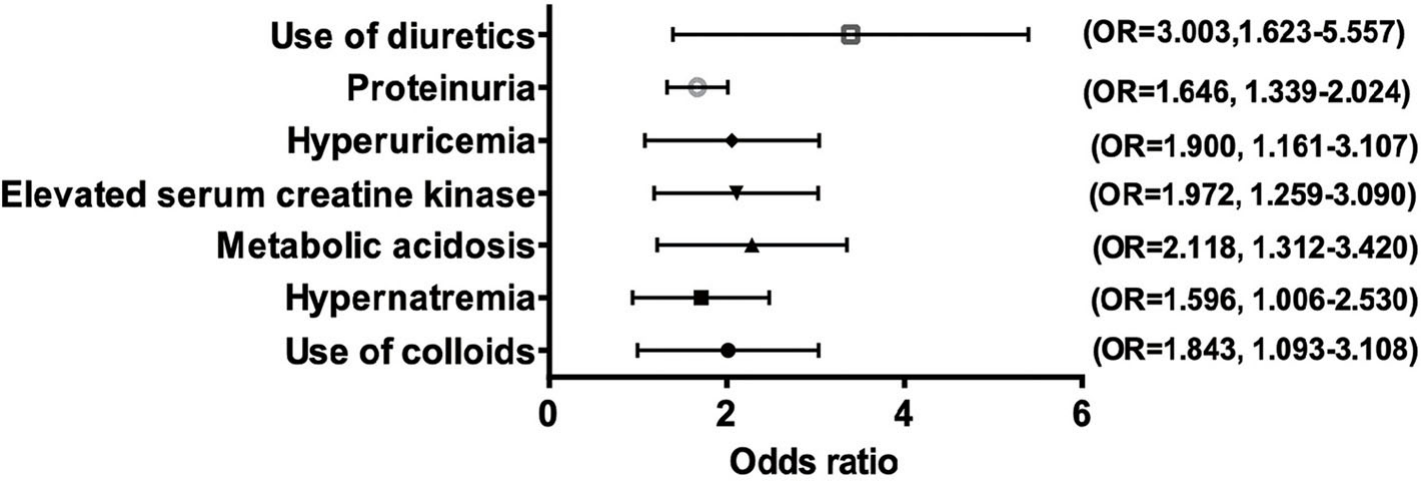
Multivariate-adjusted ORs of risk factors (*P* < 0.05) for SICH patients complicated by stage 3 AKI. Adjusted for age ≥ 65 years, male gender, mechanical ventilation, ICU admission, hypernatremia, hyperchloremia, metabolic acidosis, elevated serum creatine kinase, hyperuricaemia, elevated white blood cell counts, anaemia, proteinuria, haematuresis, and the use of medications including colloid (serum albumin), vasoactive agents, and diuretics

**Fig. 2:**
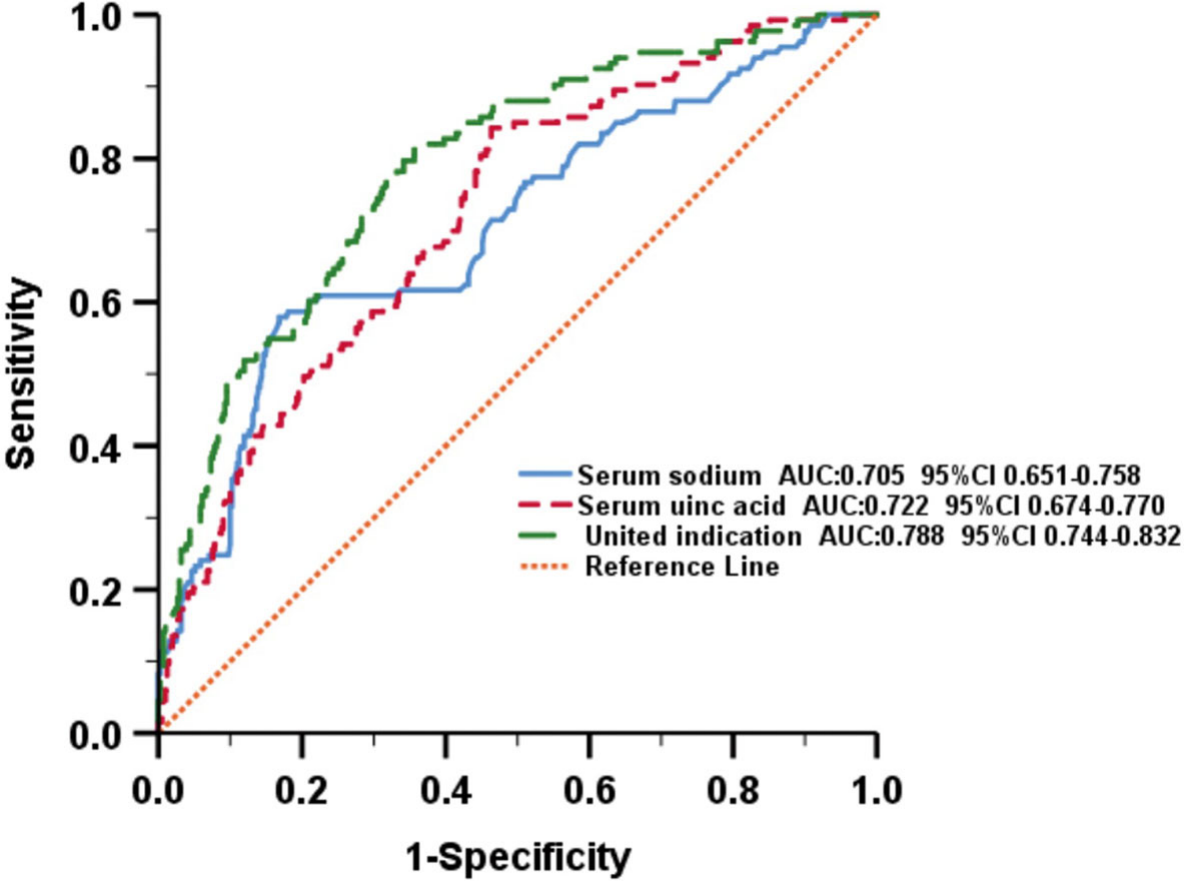
ROC curve for predicting the occurrence of stage 3 AKI. United indication: the combination of serum sodium and serum uric acid

To determine the risk factors that might contribute to stage 3 AKI, univariate logistic regression analysis was first performed by inclusion of all clinically related predictors. As presented in Table 2, significantly different variables that facilitated the occurrence of stage 3 AKI included mechanical ventilation, ICU admission, hypernatremia, hyperchloremia, metabolic acidosis, elevated serum creatine kinase, hyperuricaemia, elevated white blood cell count, anaemia, proteinuria, haematuresis, and the use of medications including colloids (serum albumin), vasoactive agents, and diuretics. Based on the results from univariate analysis and after adjustment for confounders, multivariate logistic regression analysis was implemented showing that hypernatremia (OR = 1.596, *P* = 0.047), metabolic acidosis (OR = 2.118, *P* = 0.002), elevated serum creatine kinase (OR = 1.972, *P* = 0.003), hyperuricaemia (OR = 1.900, *P* = 0.011), proteinuria (OR = 1.646, *P* < 0.001), the use of colloids (OR = 1.843, *P* = 0.022) and the use of diuretics (OR = 3.003, *P* < 0.001) were all independent risk factors for stage 3 AKI as their odds ratios were all greater than 1 and of statistical significance (Fig. 1). Of note, higher serum sodium levels and hyperuricaemia were associated with the occurrence of stage 3 AKI as well. Having identified the risk factors, we next performed the AUC-ROC curve analysis to calculate the sensitivity and specificity of the optimal cut-off values of individual independent factors to more accurately predict the occurrence of stage 3 AKI. As shown in Fig. 2, a cut-off value of 151.25 mmol/L serum sodium concentration predicted the occurrence of stage 3 AKI (AUC = 0.705; 95% CI, 0.651–0.758; sensitivity 57.9%; specificity 83.2%), whereas serum uric acid levels of 307.25 μmol/L predicted occurrence of stage 3 AKI (AUC = 0.722; 95% CI, 0.674–0.770; sensitivity 84.2%, specificity 53.2%). Nevertheless, the AUC of serum sodium and uric acid combination reached 0.788 (95% CI, 0.744–0.832; sensitivity 80.5%; specificity 64.4%), suggesting that the combination of these two variables better predicts the occurrence of stage 3 AKI than either single variable alone. Note that the ROC curves and corresponding AUC of less than 0.7 for haemoglobin (AUC = 0.589), serum creatine kinase (AUC = 0.657), and serum bicarbonate (AUC = 0.602) were excluded from Fig. 2.

**Table 2:** Univariate analysis of risk factors for deterioration to AKI stage 3 in SICH patients complicated with AKI

Characteristics	OR	95%CI	*P* value
**Demographics**			
Age ≥ 65 years	1.081	0.714–1.637	0.712
Male	1.206	0.790–1.840	0.385
**Medical history**			
Diabetes	1.546	0.951–2.512	0.079
Hypertension	1.135	0.762–1.691	0.532
ICU admission	1.589	1.034–2.470	**0.035**
Mechanical ventilation	2.540	1.649–3.911	**<0.001**
**Medication used**			
Mannitol	1.025	0.661–1.591	0.912
NSAID	1.057	0.701–1.591	0.791
Colloids	2.186	1.377–3.472	**0.001**
Vasoactive drugs	1.703	1.109–2.611	**0.015**
Diuretics	3.694	2.136–6.389	**<0.001**
Aminoglycosides	1.228	0.694–2.171	0.48
**Laboratory data**			
Serum sodium (normal)	0.214		**<0.001**
Hypernatronemia*	1.719	1.101–2.685	**0.017**
Hypernatronemia†	2.42	1.186–4.936	**0.015**
Hypernatronemia‡	8.061	3.615–17.971	**<0.001**
Hyperchloremia	1.994	1.340–2.996	**0.001**
Metabolic acidosis	2.186	1.447–3.301	**<0.001**
Hypoproteinemia	1.028	0.689–1.534	0.893
Elevated serum creatine kinase	2.962	1.981–4.428	**<0.001**
Hyperuricemia	3.074	1.990–4.748	**<0.001**
Elevated white blood cell count	1.716	1.107–2.662	**0.016**
Anemia	1.637	1.081–2.478	**0.02**
Proteinuria	1.82	1.505–2.200	**<0.001**
Hematuresis	1.406	1.219–1.662	**<0.001**

*ICU admission* intensive care unit admission;Serum sodium (normal): serum sodium concentration ≤ 145 mmol/L; Hypernatronemia*: serum sodium concentration > 145 mmol/L & ≤ 160 mmol/L; Hypernatronemia†: serum sodium concentration > 160 mmol/L & ≤ 170 mmol/L; Hypernatronemia‡: serum sodium concentration > 170 mmol/L.

### Short-term prognosis in SICH patients complicated by AKI

**Fig. 3:**
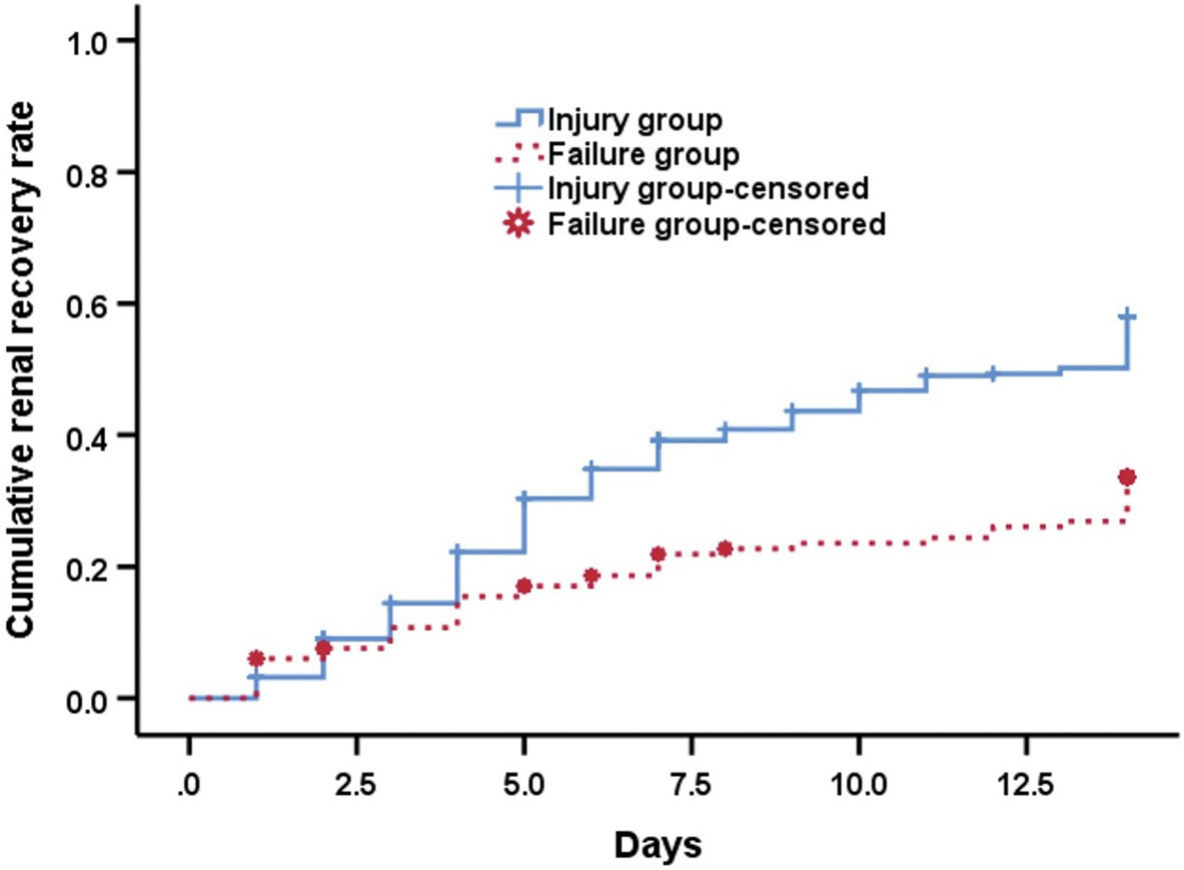
Kaplan-Meier curve of the cumulative renal recovery rate at 14 days according to stage of AKI

**Fig. 4:**
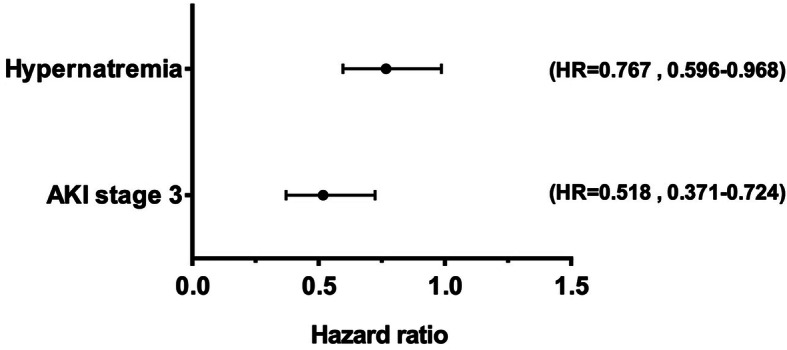
Multivariate-adjusted HRs of risk factors (P < 0.05) for renal recovery in SICH patients complicated by AKI at 14 days. Adjusted for age ≥ 65 years, male gender, mechanical ventilation, ICU admission, stage 3 AKI, elevated serum creatine kinase, hypernatremia, hyperchloremia, and the use of diuretics

Next, we attempted to assess the effect of AKI severity on the clinical outcome of SICH. Hospital length of stay (LOS) was indeed significantly longer for SICH patients with stage 3 AKI compared to those with stage 1 and 2 AKI (mean LOS of 25 days vs 19 days, respectively, *P* < 0.01). By the 14-day follow-up after admission, SICH patients with more severe AKI (stage 3) were less likely to recover renal function than patients with stage 1 and 2 AKI [*N* = 39 (39/133, 29.32%) for Failure group vs *N* = 193 (193/410, 47.07%, *P* = 0.004)]. As assessed by Kaplan-Meier curve and log-rank test, SICH patients in the Failure group had a significantly reduced renal recovery rate compared to those in the Injury group during the 14-day period after AKI (χ^2^ = 19.216, *P* < 0.001) (Fig. 3). Defining renal recovery as a terminal event, the log-rank test was then performed to evaluate significant factors affecting renal recovery in SICH patients complicated by AKI. Results showed that mechanical ventilation (χ^2^ = 6.681, *P =* 0.01), ICU admission (χ^2^ = 4.782, *P =* 0.029), hypernatremia (χ^2^ = 7.897, *P =* 0.005), hyperchloremia (χ^2^ = 4.664, *P =* 0.031), elevated serum creatine kinase (χ^2^ = 6.814, *P =* 0.009), and the use of diuretics (χ^2^ = 4.488, *P =* 0.034) were significantly associated with renal function recovery (Table 3). After adjustment for confounding factors, multivariate Cox regression analysis identified hypernatremia (HR = 0.767, 95% CI, 0.596–0.986) and the occurrence of stage 3 AKI (HR = 0.518, 95% CI, 0.371–0.724) as the two significant contributors to the recovery of renal function in SICH patients complicated by AKI over 14 days after hospital admission (Fig. 4).

**Table 3:** Log-Rank test was to identify the significant factors for renal recovery in SICH patients complicated with AKI

Characteristics	Chi-Square(χ2)	*P*
**Demographics**		
age ≥ 65 years	0.347	0.556
Male	2.152	0.142
**Medical history**		
Diabetes	1.404	0.236
Hypertension	0.853	0.356
ICU admission	4.782	**0.029**
Mechanical ventilation	6.618	**0.010**
**Medication used**		
Mannitol	0.000	1.000
NSAID	0.391	0.532
Colloids	2.084	0.149
Vasoactive drugs	0.217	0.641
Diuretics	4.488	**0.034**
Aminoglycosides	1.053	0.305
**Laboratory data**		
Anemia	0.452	0.501
Hypernatronemia	7.897	**0.005**
Hyperchloremia	4.664	**0.031**
Metabolic acidosis	3.247	0.072
Hypoproteinemia	0.045	0.831
Elevated serum creatine kinase	6.814	**0.009**
Hyperuricemia	0.854	0.355
Elevated white blood cell count	0.135	0.713

## Discussion

AKI is a common complication of SICH and is significantly associated with a higher rate of mortality in patients who survive SICH longer than 2 days [2]. While the mechanisms underlying the effect of SICH on induction of renal dysfunction remain to be fully illuminated, it is generally thought that acute brain injury can lead to neurohormonal changes that directly affect renal function by increasing renal sympathetic nervous system activity to alter renal blood flow and glomerular filtration and by altering vasopressin release, leading to changes in sodium and water balance [14]. The majority of previous studies reporting a link between SICH and AKI have been focused on the incidence and risk factors for development of AKI after an episode of SICH. This study is the first to evaluate factors for the occurrence of the stage 3 AKI in SICH patients and to identify prognostic factors for short-term renal recovery. Our study suggests that hypernatremia and hyperuricaemia may be associated with stage 3 AKI in SICH patients and that hypernatremia and the occurrence of stage 3 AKI predicted poor short-term prognosis in the setting of AKI-complicated SICH.

Patients with SICH often have electrolyte imbalances in the body, and elevation of blood sodium levels often predicts a poor prognosis [15]. The cause of hypernatremia may be related to the early use of hypertonic dehydrating agents and diuretics, consequently leading to dysregulated water and sodium metabolism in the body. Meanwhile, SICH can cause hypothalamic dysfunction of the posterior lobes of the pituitary gland, affect osmotic receptors in the brain and impairing secretion of vasopressin, together resulting in elevated serum sodium levels [16]. A retrospective study analysing 152 patients admitted to the ICU found that hypernatremia was an independent risk factor both for development of AKI and for mortality of ICU patients [17]. More specifically, Kumar et al found that sodium exposure was significantly associated with AKI risk in patients with subarachnoid haemorrhage [18]. For each 1 mEq/L increase in serum sodium levels, the hazard of developing AKI was increased by 5.4%. Our results further extended this finding by demonstrating that hypernatremia is an independent risk factor for the occurrence of stage 3 AKI in patients with SICH complicated by AKI and that hypernatremia is one of the potential clinical predictors for the occurrence of stage 3 AKI with an optimal cut-off value of 151.25 mmol/L.

It has been frequently reported in recent years that hyperuricaemia is also an independent risk factor for AKI [18, 19]. Animal studies suggest that serum uric acid may be involved in a series of processes leading to AKI, such as renal vasoconstriction, endothelial dysfunction, the inflammatory response and oxidative stress [19, 20]. A study by Lapsia et al revealed that serum uric acid (SUA) levels greater than 7 mg/dL were associated with increased risk for AKI, increased length of hospital stay and a longer duration of mechanical ventilation support [21]. In addition, in an animal model of acute renal failure caused by cisplatin, high uric acid led to increased inflammation of the kidney tissue, further aggravating kidney damage, and treatment that reduced uric acid levels may reverse this damage [22]. Consistently and furthermore, our results found that hyperuricaemia is an independent risk factor and potential predictor for the occurrence of stage 3 AKI in SICH patients with an optimal cut-off value of 307.25 μmol/L.

The effect of diuretics on outcomes of critically ill patients with AKI is still controversial. In clinical practice, diuretics are often used in critically ill patients to treat and prevent fluid overload. One study found that diuretic therapy was more effective at reducing clinical symptoms but at the cost of decreased renal function in patients with acute heart failure [23]. Accumulating evidence indicates that diuretics may not be beneficial for the prevention and treatment of AKI. The use of furosemide has proven ineffective in preventing AKI after cardiac surgery and increases the risk of contrast reagent-induced nephropathy [24]. An epidemiological study suggested that use of loop diuretics could increase mortality in critically ill patients with AKI [25]. A meta-analysis also showed that diuretics neither reduce the mortality of AKI patients nor promote recovery of renal function [26]. Therefore, the KDIGO guidelines recommend not using diuretics to prevent and treat AKI. Unfortunately, few studies have reported on the influence of diuretics with respect to outcomes in different subsets of AKI patients. Shen et al. [25] found that the association between poor outcomes and furosemide was more frequently reported in cohorts with higher SCr (> 3.0 mg/dl), while insignificant in patients with mild AKI (< 2.0 mg/dl) [27]. In our study, we found that the use of diuretics (OR = 3.003, *P* < 0.001) constituted a greater risk for the occurrence of a more severe stage of AKI in SICH patients complicated by AKI after adjusting for age ≥ 65 years, male gender, mechanical ventilation, ICU admission and other confounding factors.

Currently, there is no standardized time window for evaluation of short-term renal function recovery after AKI, but most studies have used 7–28 days after the onset of AKI [28, 29]. In compliance with the regulations of our hospital on the length of stay allowed for SICH patients, we opted to choose day 14 after AKI as the recovery assessment time. Our results showed that both hypernatremia and the occurrence of stage 3 AKI were not conducive to recovery of renal function in patients with SICH complicated by AKI. Thus, early correction of hypernatremia and avoiding the occurrence of stage 3 AKI may minimize harm to the kidneys in this subset of patients and improve prognosis.

It should be noted that some limitations exist in our study. First, due to the retrospective design of these analyses, we could not control for all confounding parameters or obtain all factors that might have affected our results. There is no direct evidence which shows that these factors are associated with the efficacy of hypernatremia and diuretics in SICH patients with AKI. Thus, the risk of confounding factors should be taken into consideration when interpreting these results. Second, we were limited by the length of hospital stay after AKI and were unable to use a better endpoint, such as “death”, for survival analysis. Third, we assessed prognosis over a short-term period only. As such, prospective studies with larger sample sizes are needed to verify these findings.

## Conclusions

In conclusion, the occurrence of stage 3 AKI in SICH patients is associated with hypernatremia, metabolic acidosis, elevated serum creatine kinase, hyperuricaemia, anaemia, proteinuria, and the use of diuretics. Either hypernatremia or hyperuricaemia is a potential clinical indicator for predicting the occurrence of stage 3 AKI, while the combination of these two parameters is of more predictive value. Compared to SICH patients complicated by stage 1 and stage 2 AKI, the length of hospital stay and kidney recovery is significantly increased in patients with stage 3 AKI. In addition, renal recovery rate during the 14-day period after AKI is lower in SICH patients complicated by stage 3 AKI and hypernatremia.

## Data Availability

The datasets used and/or analysed during the current study are available from the corresponding author on reasonable request.

## Abbreviations

AKI: Acute kidney injury
SICH: Spontaneous intracerebral haemorrhage
ROC: Receiver operating characteristic
AIS: Acute ischaemic stroke
KDIGO: Kidney Disease: Improving Global Outcomes
CKD: Chronic kidney;disease
SCr: Serum creatinine
NSAID: Non-steroidal Anti-inflammatory drug
ICU: Intensive care unit
LOS: Length of stay
SUA: Serum uric acid
